# Effects of Kitchen Cooking Height on Upper Limb Muscle Activation, Posture, and Perceived Discomfort of Chinese Older and Young Women

**DOI:** 10.3390/s24217056

**Published:** 2024-11-01

**Authors:** Ye Li, Le Chang, Fan Zhang

**Affiliations:** College of Materials Science and Technology, Beijing Forestry University, Beijing 100083, China; jasmineye@bjfu.edu.cn (Y.L.); zhangfan1976@163.com (F.Z.)

**Keywords:** kitchen cooking height, age difference, muscle activation, posture analysis, rating of perceived discomfort, comfort

## Abstract

Inappropriate kitchen cooking height may lead to uncomfortable and muscle fatigue. This study aims to compare the effects of kitchen cooking height on upper limb muscle activation, posture, and perceived discomfort among different age groups. Fifteen older women and fifteen young Chinese women each completed three consecutive 20 s simulated cooking tasks at five different heights. Surface electromyography, motion capture, and Borg CR10 scale were used to measure muscle loading. Results showed that the main power muscles of the cooking task were the anterior deltoid, brachioradialis, and biceps brachii. The higher muscle contribution rate of biceps brachii and triceps brachii was found in the younger group compared to the older group (*p* < 0.05). Muscle activation of the anterior deltoid (different in 1.28–2.87%), pectoralis major (different in 1.43–1.69%), and erector spinae (different in 0.6–1.21%), as well as right shoulder abduction (different in 5.91°–7.96°), were significantly higher in older group than in young group (*p* < 0.05). Muscle activation of the anterior deltoid and right shoulder abduction decreased significantly with decreasing height (*p* < 0.05). A height of 200–250 mm below the elbow was considered a more comfortable cooking height for both age groups. This provides data to support the design of cabinet sizes.

## 1. Introduction

The trend of population aging has become an important issue that China and most countries in the world need to face. Musculoskeletal disorders (MSDs) exhibit an age-dependent prevalence, with women being disproportionately affected [1,2]. It has been reported that physical work capacity declines by an average of 20% between the ages of 40 and 60 due to decreases in aerobic and musculoskeletal capacity [3]. These declines can lead to a reduced ability to work and, consequently, increased work-related injuries and illnesses. The kitchen, as a central activity area in the home, plays a vital role in the daily routines of older adults [4]. Most Chinese older adults prefer to continue to live at home or receive community-based older adult care services [5]. Studies have demonstrated that older adults in the kitchen are prone to problems with reaching, bending, flexibility, and vision [6]. The primary risk factors associated with MSDs in kitchen workers include lifting heavy weights, repetitive upper limb movements, prolonged standing, and forward trunk flexion [7,8,9]. Therefore, ergonomic kitchen design for older adults is essential to facilitate independent living and operation in their later lives. Several studies have explored factors influencing these risks, such as knife design [10,11], workstation ergonomics [12], and the use of exoskeletons or collaborative robots [13].

One key aspect of ergonomic design is ensuring appropriate dimensions for the kitchen environment, such as the height of the cooking surface and countertop. Excessively high kitchen countertops can lead to shoulder issues for shorter users, caused by prolonged upper limb lifting. Some studies have employed anthropometric measurements [14] and subjective evaluations [15] to determine optimal heights for kitchen sinks, work surfaces, and stoves. In Ward’s study [16], muscle activation in lower limb muscles—such as the quadriceps, gastrocnemius, biceps femoris, and erector spinae—was objectively measured using surface electromyography (sEMG) to assess suitable countertop heights for adult women in the United Kingdom. To our knowledge, no study has yet quantified how the height of kitchen cooking surfaces affects musculoskeletal loading in older adults, particularly regarding differences between older and young adults in kitchen tasks. Older adults often experience significant differences in joint mobility due to arthritis and other musculoskeletal disorders, making the impact of kitchen work surfaces on their comfort and posture especially pertinent [17].

Numerous studies have quantitatively assessed muscle loading by measuring changes in muscle activation using surface electromyography (sEMG), a technique that measures the electrical activation associated with muscle contraction and relaxation [18], particularly in studies involving older adults. However, most research on muscle activation changes in this population has primarily focused on the lower limbs [19,20,21,22], emphasizing fall prevention, while upper limb studies have been less prevalent. Nonetheless, a few articles have explored upper limb muscle characteristics in older adults, such as examining the relationship between the height of hanging operating [23], the height and distance of armchairs during sit-to-stand transitions [24], and upper limb muscle activation, which has significantly inspired our research. In kitchen cooking activities, discomfort is primarily reported in the neck, shoulders, and lower back [25,26,27]. The upper limb muscles, including the trapezius, deltoids, biceps, and triceps, are essential power muscles during kitchen tasks and play a crucial role in operational comfort [28,29].

Furthermore, there is a strong connection between body posture and muscle loading. Motion capture technology effectively assesses user comfort by capturing data on body posture and has been widely employed in evaluating working postures in various settings, including offices [30,31], industrial environments [32], and even in robotic applications [33]. Some researchers have integrated myoelectric and kinetic tracking techniques to study comfort. For example, Zhou examined the effects of different loads and retrieval postures on muscle activation and joint range of motion when older adults retrieve objects from high locations [34]. Similarly, Komisar investigated how handrail height influences the timing and speed of reach-to-grasp balance reactions during slope descent in both young and older adults [35]. Jakob analyzed the effects of working height and manipulated weights on body posture and muscular activation among milking parlor operatives [36].

Therefore, this study aimed to comprehensively characterize the effect of kitchen cooking height on musculoskeletal loads across different age groups by combining sEMG and motion capture techniques to determine the optimal countertop height. Muscle activation was measured by sEMG, body joint angles were assessed through motion capture, and overall and localized perceived discomfort was evaluated using the Borg CR10 scale. The relationship between kitchen cooking height and these indicators was then analyzed, focusing on differences between older and young adults. Ultimately, the findings were used to propose optimal cooking heights for both age groups. The study hypothesized that changes in kitchen cooking height would affect these physiological indices in participants and anticipated variability in the changes between older and young adults.

## 2. Materials and Methods

### 2.1. Subjects

The subjects were divided into an older group (OG) and a young group (YG). 15 older Chinese women and 15 young Chinese women volunteered to participate in this study (Table 1). Subjects were limited to females to exclude the influence of the gender factor on the results of the experiment. All participants had kitchen cooking experience. The project was reviewed by the Human Study Ethics Committee of Beijing Forestry University (BJFUPSY-2024-035). Each subject signed a consent form and completed a written screening survey to ensure that they had no significant musculoskeletal disorders and were right-handed, and none had engaged in strenuous exercise the week before this experiment.

### 2.2. Experimental Measurements

#### 2.2.1. sEMG

sEMG signals were used to quantify muscle activation in participants. An 8-channel sEMG signal measurement and acquisition system was used in the experiment (Kingfar Technology Inc., Beijing, China). The sEMG signals were sampled at a frequency of 1000 Hz with a band-pass filter tuned at 10–500 Hz. The wearable sEMG sensors were used for the sEMG signal acquisition, and the ErgoLAB human–machine synchronous physiology cloud platform was used to record, process, and output the sEMG signal data.

**Figure 1:**
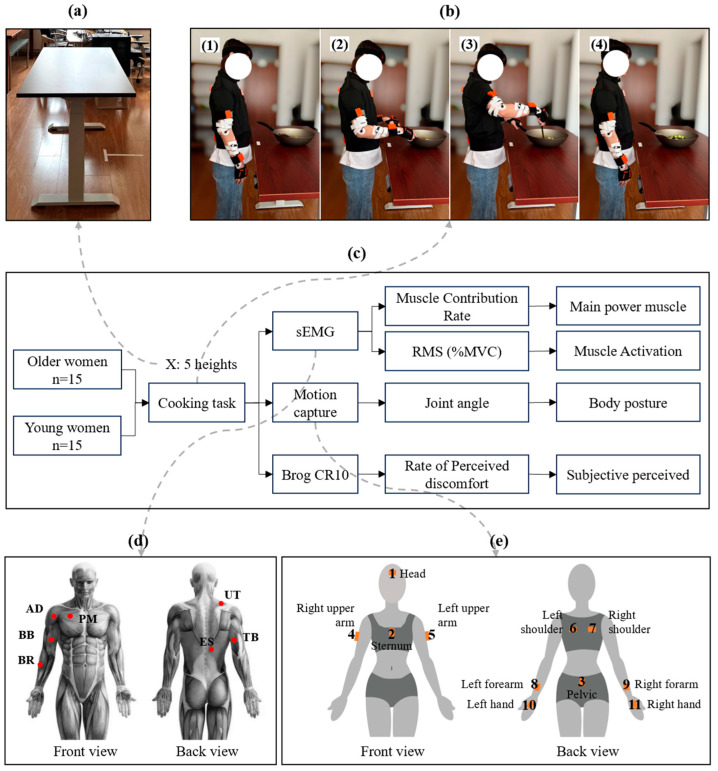
Overview of the experimental setup. (**a**) Laboratory environment. (**b**) Demonstration of the kitchen cooking task. b1. Stand with arms down. b2. Hold the handle of the pan with the left hand and the frying spoon with the right hand. b3. Stir fry the cucumber slices. b4. Stop operation and stand with arms down. (**c**) Experimental setup. (**d**) Positions of sEMG sensors. (**e**) Positions of motion capture sensors.

Seven muscles of the right upper limb and trunk related to the manipulation movements were selected for testing because the test movements were mainly the right side of the body firing and the height change mainly affected the muscle activation of the upper body. The muscles measured in this experiment were the upper trapezius (UT), pectoralis major (PM), anterior deltoid (AD), biceps brachii (BB), triceps brachii (TB), brachioradialis (BR), and erector spinae (ES) (Figure 1d). The electrode placement locations followed recommendations from the SENIAM Project [38].

Disposable electrode pads were used to connect the sEMG sensors, where the center-to-center distance of the electrode pads was approximately 20–30 mm parallel to the direction of the muscle fibers [39]. Before attaching the electrodes, the skin should be wiped with alcohol to reduce the impedance [40].

In addition, the maximal volitional contraction (MVC) of the muscle was measured to normalize the EMG amplitude (%MVC). Participants were required to perform three 5 s maximal efforts in different arm and body positions [41]. The muscle contribution rate of each muscle was calculated from the iEMG values in the sEMG signals, which were used to determine the main power muscles under each set of tasks [42]. Normalized RMS values (%MVC) were chosen to characterize the muscle activation, with higher values indicating a greater tendency to fatigue the more forceful the muscle is [43,44].

The equations for iEMG (1), RMS (2), and muscle contribution rate (3) are as follows:

(1) $$\mathrm{iEMG}=\int_{N2}^{N1}X\left(t\right)\mathrm{dt}$$

(2) $$\mathrm{RMS}=\sqrt{\frac{1}{N}\int_{i=1}^{N}\mathrm{EMG}{\left(i\right)}^{2}}$$

η = (I_n_/(I_1_ + I_2_ + I_3_ + · · · + I_n_)) × 100% (n = 1, 2, 3, 4, 5, 6, 7) (3)

#### 2.2.2. Motion Capture

The experiment used a motion capture system to acquire data on participants’ body postures (Xsens Technologies B.V., Enschede, The Netherlands). The system adopts inertial sensing technology to acquire body posture data such as joint angles under different postures of the subject in real time. The sensors were attached to the corresponding positions on the body in the form of Velcro. This experiment focused on upper limb postural characteristics. Sensors were worn at 11 upper body points, including forehead, breast, left back, right back, left upper arm, right upper arm, left wrist, right wrist, left hand, right hand, and hip (Figure 1). Calibration of the equipment was necessary before conducting the formal experiments.

Joint angles acquired using the motion capture system are used for posture analysis. Some methods provided a risk assessment regarding biomechanical postural load such as ISO11226-2000 [45] and Rapid Upper Limb Assessment (RULA) [46]. They were applied to evaluate posture data in the study. It was primarily analyzed for upper limb joint angles, including head, trunk, and right arm joint angles (Appendix A).

#### 2.2.3. Borg CR10 Scale

The rating of perceived discomfort (RPD) was scored on the Borg CR10 scale ranging from 0 (nothing at all) to 10 (very, very strong). This scale includes assessments of overall discomfort as well as specific body parts, including the neck, shoulders, upper arms, forearms, wrists, and lower back [47].

### 2.3. Experimental Procedures

This study was conducted in a laboratory setting (Figure 1). Kitchen cooking surfaces at different heights were simulated by adjusting the height of the table. sEMG signals and body posture data were acquired in different settings. The test height was based on the subject’s elbow height, which is considered to be a key reference for determining work scales [48,49]. Following pre-experimental testing, the final test heights were established at five different levels (Table 2).

The experimental task was designed to simulate a stir-frying action, commonly seen in Chinese cooking. Participants performed the stir-frying task using cucumber slices. Each subject repeated the cooking task three times for 20 s at each height, with the cooking process standardized (Figure 1b). The task consisted of the following phases: (1) 0–5 s is the starting phase, where subjects stand with their hands hanging naturally; (2) 5–10 s is the ready phase, during which the subject holds the wok handle with their left hand and the spatula with their right hand; (3) 10–30 s is the cooking task phase, where subjects tossed the cucumbers in the pan according to their habits. This process was repeated three times for each height.

The experimental protocol included the following sections: (1) anthropometric measurements, (2) device wear and calibration, (3) kitchen cooking task, (4) evaluation of RPD, and (5) MVC measurements. Participants completed the cooking task at five different heights. After each height test, they filled out the RPD scale, followed by a 5 min break. The total duration of the test for each subject was less than one hour.

### 2.4. Statistical Analysis

Statistical analysis of muscle activation, joint angles, and RPD was performed using SPSS v.27.0 (IBM Corp., Armonk, NY, USA). Descriptive statistics were expressed as mean, standard deviation, and median. sEMG signals and body posture data were recorded from participants. Data from 2 to 18 s of a sustained 20 s movement were intercepted and analyzed, and the mean of the three repetitions at each height was calculated. Muscle activities, joint angles, and RPD were analyzed in this study.

The Shapiro–Wilk test was used to determine the normality of the data before selecting the appropriate method of analysis. Spearman’s test was used to test the correlation between muscle activities, joint angles, and table height. The effect of table height on muscle activation and joint angles under different tasks was assessed using repeated measures analysis of variance (ANOVA). Significant differences between the OG and YG at different heights were determined by simple effect analysis.

The Friedman M test was used to test for significant differences in RPD at different heights. Bonferroni correction was used to adjust the level of significance to explain the two-by-two comparison of the OG and YG. Mann–Whitney U test was used to test for between-group differences between the OG and YG at different heights.

## 3. Results

### 3.1. Muscle Activation Analysis

#### 3.1.1. Muscle Contribution Rate

**Figure 2:**
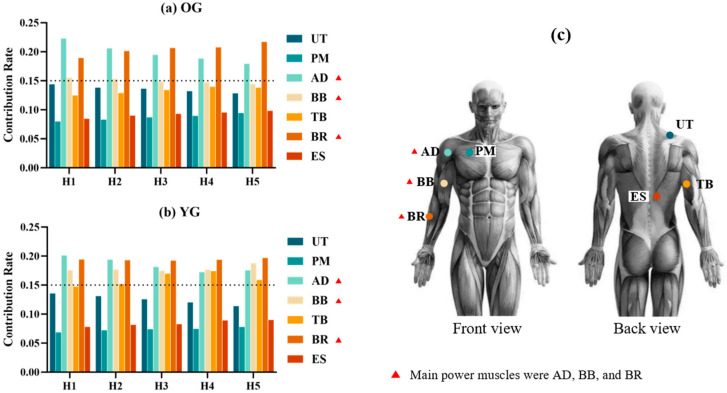
Muscle contribution rates and main power muscles in the OG and YG. (**a**) Muscle contribution in the OG. (**b**) Muscle contribution in the YG. (**c**) Muscle position. Note: The dash line indicates muscle contribution rate equal to 15%.

The muscle contribution rates can respond to subjects’ primary power muscles and force-generating patterns while performing the cooking task. As shown in Figure 2, the main power muscles involved in both groups were the AD, BR, and BB, which were used as the main focus for analyzing muscle activation. However, the YG exhibited a significantly higher contribution rate from the BB and TB compared to the OG (*p* < 0.05).

#### 3.1.2. Muscle Activation

Muscle activation was characterized using normalized RMS values (%MVC). The correlation coefficients between the RMS values and the test heights are shown in Table 3. Significant correlations were found for the RMS values of UT, AD, and ES in both groups (*p* < 0.05). In the OG, the RMS values of the UT, AD, and BB were positively correlated with height (*p* < 0.05), and the RMS values of the ES were negatively correlated with height (*p* < 0.05). In the YG, the RMS values of the UT, AD, and BR showed a positive correlation with height (*p* < 0.05), whereas the RMS values of the PM and ES exhibited a negative correlation with height (*p* < 0.05).

**Figure 3:**
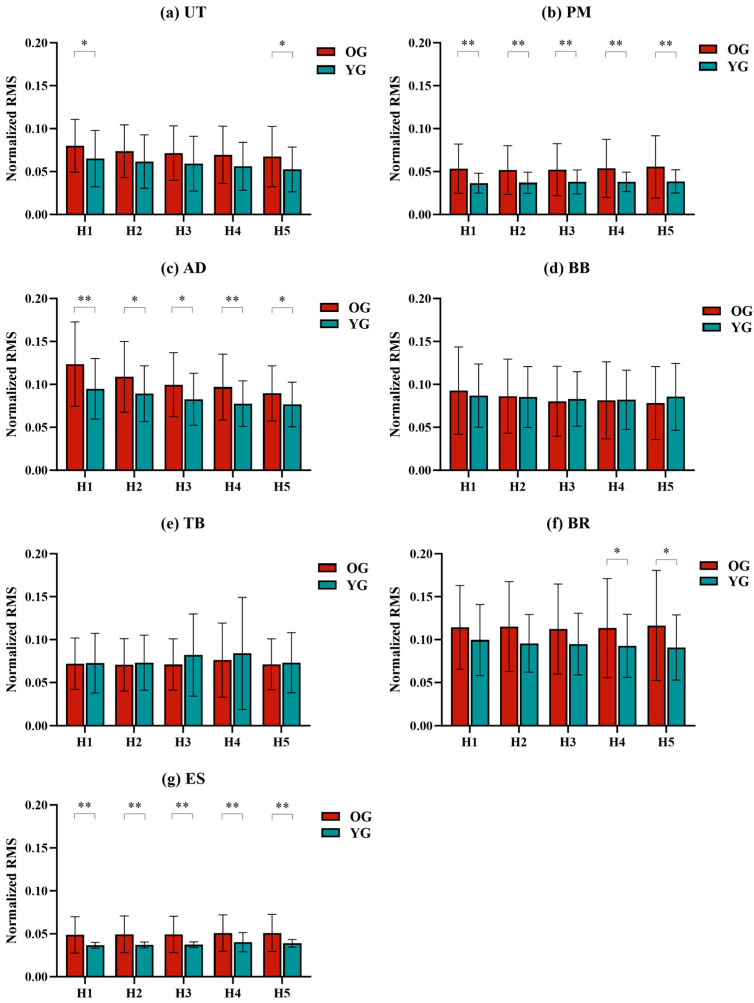
The normalized RMS values at different heights in the OG and YG. (**a**) UT. (**b**) PM. (**c**) AD. (**d**) BB. (**e**) TB. (**f**) BR. (**g**) ES. Note: *: *p* < 0.05, **: *p* < 0.01.

The ANOVA analysis of RMS values indicated a statistically significant effect of height on RMS values (*p* < 0.05) (Table 4). As shown in Figure 3, for the OG, the RMS values of the AD and BB decreased with decreasing height. However, the muscle activation of the AD and BB from H3 onwards was not significantly different from other heights in the OG. For BR, there were no significant differences between heights in the OG. In the YG, the RMS values of the AD also decreased with decreasing height, and similar to the OG, there was no significant difference in the muscle activation of the AD from H3 onwards. The RMS values of the BB and BR showed no significant differences between each height in the YG.

For the effects of test heights on other muscles’ activation, in both groups, the RMS values of the UT decreased with decreasing height. The muscle activation of AD was significantly greater at H5 compared to other heights. The RMS values of the PM, TB, and ES increased with decreasing height, but the differences between each height were not significant, except for ES, which had significantly greater muscle activation at H5 compared to other heights.

Additionally, comparisons of muscle activation between OG and YG showed statistically significant differences. The RMS values of the PM, AD, and ES were significantly greater in the OG than in the YG (*p* < 0.05). The difference in the RMS values of the PM was 1.43–1.69%, the AD was 1.28–2.87%, and the ES was 0.6–1.21%, respectively.

Overall, the sEMG analysis identified the main power muscles engaged during the cooking task and highlighted differences in muscle activation patterns between the OG and the YG. The analysis also revealed a significant effect of test height on muscle activation, with the OG showing significantly greater activation than the YG. These findings can contribute to evaluating the comfort associated with different cooking heights.

### 3.2. Posture Analysis

The body postures that did not meet the ergonomic requirements for static work (ISO11226-2000, RULA) in this experiment were neck flexion (>20°), shoulder abduction (>20°), elbow internal rotation (>90°), wrist abduction (>20°), and no significant back, low back, or hip flexion (<10°) (Appendix B). The correlation coefficients between these joint angles and test heights are shown in Table 5. The test height showed a negative correlation with neck flexion and right elbow internal rotation and a positive correlation with right shoulder abduction and right wrist abduction (*p* < 0.01).

**Figure 4:**
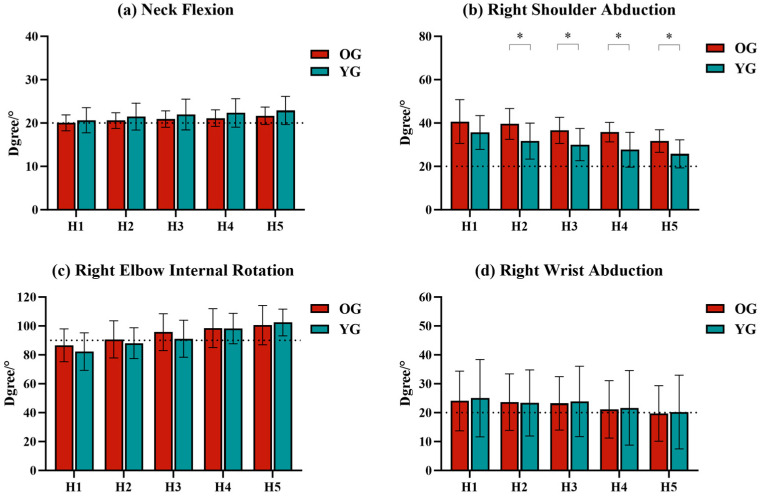
The joint angles at different heights in the OG and YG. (**a**) Neck flexion. (**b**) Right shoulder abduction. (**c**) Right elbow internal rotation. (**d**) Right wrist abduction. Note: *: *p* < 0.05. the dash line in a is 20°, the dash line in b is 20°, the dash line in c is 90°, the dash line in d is 20°.

The ANOVA analysis of joint angles showed that height had a significant effect on joint angles (*p* < 0.01) (Table 6). As shown in Figure 4, neck flexion and right elbow internal rotation gradually increased, and right shoulder abduction and right wrist abduction gradually decreased with decreasing test height. Neck flexion was within 20°–25° in both the OG and YG. Right shoulder abduction was within 20°–60° in both the OG and YG. Right elbow internal rotation was more than 90° at H2 in the OG and more than 90° at H3 in the YG. Right wrist abduction was less than 20° at H5 in the OG, whereas the right wrist abduction of the YG is greater than 20° at all heights.

Moreover, comparisons of body posture between OG and YG showed statistically significant differences. Right shoulder abduction was significantly greater in the OG than in the YG, except at H1 (*p* < 0.05), with a difference ranging from 5.91° to 7.96°.

To summarize, the analysis of body postures identified specific joint angles associated with non-ergonomic static postures during cooking tasks. It was found that cooking height significantly influenced these joint angles, and differences in operating postures were observed between the OG and the YG. The combination of sEMG and motion capture indicators provides a comprehensive assessment of cooking height comfort, highlighting differences between older and young populations.

### 3.3. RPD Analysis

In addition to the objective results, comfort at different heights was also assessed using subjective discomfort scores. As shown in Table 7, the RPD of overall and body parts was significantly different in the OG at different test heights, except for the forearm (*p* < 0.05). The YG had significant differences in the neck and low back (*p* < 0.05).

**Figure 5:**
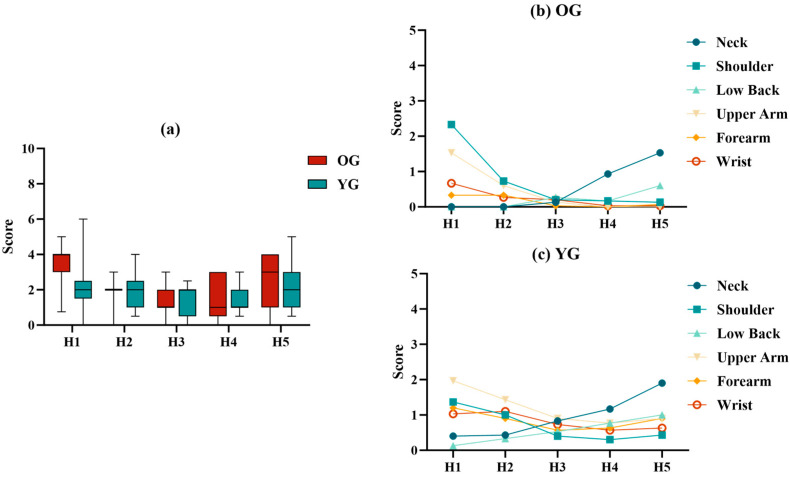
RPD of the overall body at different heights for OG and YG. (**a**) RPD of the overall body. (**b**) RPD of body parts in the OG. (**c**) RPD of body parts in the YG.

In the RPD of the overall body, as shown in the box plot (Figure 5a), H3 and H4 had the lowest scores in the OG, while the YG had the lowest score at H4. The main discomfort areas in the OG were the shoulder, neck, and upper arm (Figure 5b), whereas in the YG, they were the upper arm, neck, and shoulder (Figure 5c). The RPD in the upper arm, shoulder, and wrist gradually decreased with decreasing height, while the RPD in the neck and low back gradually increased.

## 4. Discussion

Due to the lack of quantitative data on musculoskeletal loading in older adults during kitchen activities, this study combined sEMG and motion capture to investigate the effects of cooking height on muscle activation, posture, and perceived discomfort in older and young adults in terms of kitchen cooking tasks in their daily lives. It was found that changes in kitchen cooking height significantly affected muscle activation and body posture. As the cooking height decreased, the muscle activation of the major muscle, AD, gradually decreased, neck flexion and right elbow internal rotation gradually increased, and right shoulder abduction and right wrist abduction gradually decreased in both the OG and YG. Furthermore, this study compared differences between older women and young women. The YG exhibited a significantly higher contribution rate from the BB and TB than the OG. Muscle activation of the PM, AD, and ES were significantly higher in the OG compared to the YG, and right shoulder abduction was significantly higher in the OG. Both OG and YG consider cooking heights of H3 or H4 (200–250 mm below the elbow) to be the most comfortable.

Our findings reveal that older and young women have different force generation patterns in kitchen cooking tasks. It was found that there was a higher muscle contribution rate of the BB and TB in the YG compared to the OG, and right shoulder abduction was significantly higher in the OG than in the YG in this study. This indicated that the older women tended to exert force on the shoulder, while the young women tended to exert force on the upper arm. Some studies have suggested that older adults adopt a more “economic” motor strategy, which reduces the biomechanical load on the upper extremity [50]. Norheim, in his study of arm kinematics during hammering movements, also found that older workers seemed to use a less biomechanically demanding movement strategy [51]. This may be related to the cooking experience and habits of older and young people, potentially contributing to different areas of muscle fatigue in different age groups.

This study concluded that older women should be more aware of shoulder muscle fatigue and pain when performing kitchen cooking tasks compared to young women. Jonsson pointed out that for tasks longer than one hour, the average muscle activation should not exceed 10% [52]. It was found that the muscle activation of the BR and AD in the OG exceeded this recommended threshold, meaning that older women were more likely to experience muscle fatigue in the shoulder, forearm, and wrist areas during prolonged kitchen cooking. Additionally, it also was found that the muscle activation of the PM, AD, and ES was significantly higher in the OG than in the YG. Arjunan and Kumar [53] found that as individuals age, the complexity of sEMG decreases, while the variability in muscle contractility increases. Older adults require more time and effort to complete tasks compared to young adults [54,55]. Qin et al. observed a more pronounced and consistent decrease in EMG power frequency among older adults, indicating reduced resistance to fatigue in the trapezius and upper deltoid muscles during repetitive tasks [56,57]. Furthermore, in this experiment, the body postures that did not meet the ergonomic requirements for static work were neck flexion, shoulder abduction, elbow internal rotation, and wrist abduction. It also confirmed that WMSDs in kitchen workers commonly occur in the body parts of the shoulder, neck, low back, and wrist. In comparison, there was more shoulder discomfort feedback from the OG and more upper arm discomfort feedback from the YG, which may be related to the prevalence of shoulder osteoarthritis and rotator cuff disease in the older population [58].

Despite different power-up patterns and muscle activation between OG and YG, both considered H3 and H4 as more comfortable cooking heights. In sEMG results, the muscle activation of the AD gradually decreased with decreasing test height, and there were no significant differences between the muscle activation at the two heights H3 and H4 and they were significantly lower than at H2 in both the OG and YG. This indicates that H3 and H4 are indeed the heights at which the upper limb muscles are more energy efficient. Studies have shown that the lower the level of shoulder abduction, the lower the muscle activation of the AD [59]. In our research, shoulder abduction decreased as height decreased, reducing muscle activation and making lower cooking surfaces more comfortable. Additionally, the muscle contribution rate and muscle activation of TB showed an increasing trend with decreasing height, indicating a shift in muscle force from the forearm to the upper arm. Previous studies have shown that complex tasks lead to a redistribution of muscle forces, with multiple muscles synergistically participating [60,61]. In addition, the muscle activation of the ES showed an increasing trend with decreasing height, indicating the presence of ES stretching (bending at the low back), and the muscle activation at H5 was significantly higher than at H3 in both the OG and YG. It has been reported that low back pain among cooks has become a major source of occupational health problems [62]. However, in our experiments the muscle activation of ES was low and none of the trunk flexion exceeded 10°. It was considered that the low back discomfort produced by kitchen workers may be related to prolonged standing rather than inappropriate cooking height [63]. The change in neck flexion angle with countertop height was small within 3°. The countertop height decrease was able to reduce discomfort in the upper arms, shoulders, and wrists, but there was a gradual increase in discomfort in the neck and low back. This also corresponds to the objective results of the muscle activation and postural analysis. Subjects reported lower localized ratings at H3. By comprehensive analysis, H3 or H4 is a more comfortable cooking height both for older and young women.

In conclusion, it was considered that the cooking height should be 200–250 mm below the elbow for both older and young women. The average elbow height of China’s adult females aged 61–70 years old is 939 mm, and the average elbow height of China’s adult females aged 25–35 years old is 973 mm (GB/T 10000-2023). Based on the 32 mm system cabinet system, it was considered suitable for older women that the height of the cooking countertop should be 704 mm or 736 mm (689–739 mm). The height of the cooking countertop suitable for young women should be 736 mm or 768 mm (723–773 mm). Numerous studies have been conducted on kitchen height in the past. Pekkarinen concluded that the shoulders would be more relaxed during cutting and stirring when located at a height of 150–200 mm below the elbow [64]. A stove height of 746–826 mm was determined from the low back height of older adult Malaysians [65]. This was a little higher than our findings, which were considered more applicable to Chinese home kitchens and more in line with Chinese women’s cooking habits and power patterns. In addition, this study is a small sample experiment, and subsequent researchers can expand the sample size and narrow the height interval to conduct research based on this study. For low back discomfort caused by prolonged standing in the kitchen, low back support attachments can be considered for kitchens for older adults to mitigate the low back discomfort associated with the operation process [66]. Moreover, when interviewing older adults, people not only considered the height that needed less effort when choosing the optimal height but also took into account real-life kitchen scenarios. For example, generally, the lower the height of the countertop the less effort it takes. However, the cooking height that is too low will make it difficult to see what is on the cutting board and observe the food cooking in the pan. In addition, there is a risk of getting your face steamed by the heat from the pan. The height of the ignition and the size of the storage design of the cabinetry should be considered to determine the height of the kitchen countertop.

The main limitation of this study is that it was conducted in a small-sample laboratory setting that did not fully simulate a real kitchen environment. However, some key features of kitchen tasks, such as repetitive hand movements, brief multiple manipulations, and static postures, were reflected, providing some insight into physical risk factors and participant comfort in kitchen tasks. Therefore, the results still hold reference value for kitchen countertop height design. Future studies could investigate the effects of other operations (cutting, washing, picking, multitasking) on muscle fatigue in a real kitchen setting. This study focused only on a Chinese female population; expanding the sample to include women from diverse cultural and ethnic backgrounds and examining gender differences could provide a more comprehensive understanding of the relationship between cooking height and muscle fatigue. Additionally, as the kitchen is a high fall-risk environment, particularly for the elderly, future research could explore lower limb muscle activation and gait changes in kitchen multitasking scenarios.

## 5. Conclusions

This is the first study to investigate differences in upper limb muscles’ activation, body posture, and perceived discomfort among women of different ages under varying kitchen cooking heights. The present study revealed that cooking height and age significantly influenced these indicators. It was found that older women and young women have different muscle force patterns and body postures. The YG showed a significantly higher contribution rate from the BB and TB than the OG. Muscle activation of the AD (different in 1.28–2.87%), PM (different in 1.43–1.69%), and ES (different in 0.6–1.21%), as well as right shoulder abduction (different in 5.91°–7.96°), were significantly higher in older women than in young women. Muscle activation of the AD and right shoulder abduction decreased substantially with decreasing cooking height. Combining the indicators, 200–250 mm below elbow height was considered a more comfortable cooking height for older and young women. This study quantified differences in muscle loading at various heights in older and young women during kitchen cooking tasks, and proposed an appropriate height for kitchen cooking that meets the cooking behavior and exertion habits of Chinese women. It also provides scientific data support for cabinet manufacturers. As aging populations increase globally, understanding muscle exertion patterns across age groups can inform the design of ergonomically suitable furniture for older adults, supporting their independence in later life.

## Figures and Tables

**Table 1:** Anthropometric characteristics of participants.

	OG (*n* = 15)		YG (*n* = 15)	
	Average	Range	Average	Range
Age	66.4 ± 4.16	60–73	23.9 ± 3.6	20–31
Height (cm)	158.7 ± 6.2	151–170, P10–P99	167.6 ± 4.5	161–175, P50–P99
Elbow height (cm)	98.7 ± 5.2	92.5–109	104.8 ± 3	101–111
Weight (kg)	61.13 ± 9.36	47–80	58.17 ± 7.53	48–75

Note: the percentile of the height range cited from GB/T 10000-2023 [37].

**Table 2:** Height settings for the cooking task.

H1	H2	H3	H4	H5
H-100 mm	H-150 mm	H-200 mm	H-250 mm	H-300 mm

Note: H indicates the elbow height of the subject.

**Table 3:** The Spearman correlation of Normalized RMS values and test heights.

	UT	PM	AD	BB	TB	BR	ES
OG	1.000 **	−0.700	1.000 **	0.900 *	−0.200	−0.200	−0.900 *
YG	1.000 **	−1.000 **	1.000 **	0.400	−0.400	1.000 **	−0.900 *

Note: ** indicates a significant correlation at the 0.01 level (two-tailed). * indicates a significant correlation at the 0.05 level (two-tailed).

**Table 4:** Mean, standard deviation, and data range of normalized RMS values (%MVC) at different test heights, with repeated measures ANOVA results.

Muscle		H1		H2		H3		H4		H5		F	*p*
UT	OG	8.00 ± 3.31 ^bcde^	2.68–14.70	7.38 ± 3.31 ^a^	2.36–15.17	7.15 ± 3.42 ^a^	2.38–14.54	6.95 ± 3.58 ^a^	2.46–13.80	6.74 ± 3.78 ^a^	2.41–13.89	6.128	<0.001 **
	YG	6.51 ± 3.29 ^cde^	2.00–14.92	6.17 ± 3.10 ^e^	1.87–14.72	5.92 ± 3.19 ^a^	2.05–15.46	5.62 ± 2.79 ^a^	2.01–12.25	5.26 ± 2.61 ^abc^	1.65–12.11	4.176	0.004 **
	O-Y *p*	0.041 *		0.089		0.091		0.06		0.037 *			
PM	OG	5.34 ± 2.87	2.67–13.23	5.18 ± 2.83	2.51–12.52	5.22 ± 3.03	2.01–12.95	5.37 ± 3.38	2.01–14.04	5.55 ± 3.62	2.32–14.93	1.765	0.143
	YG	3.65 ± 1.16	2.09–6.74	3.71 ± 1.24	2.24–6.92	3.79 ± 1.40	2.22–7.94	3.80 ± 1.13	2.32–6.95	3.86 ± 1.35	2.20–7.62	0.258	0.904
	O-Y *p*	<0.001 **		0.002 **		0.005 **		0.004 **		0.004 **			
AD	OG	12.36 ± 4.92 ^bcde^	6.43–26.81	10.89 ± 4.12 ^acde^	5.08–22.91	9.97 ± 3.73 ^abe^	4.79–24.12	9.69 ± 3.84 ^abe^	4.84–23.64	8.95 ± 3.21 ^abcd^	4.42–17.91	23.373	<0.001 **
	YG	9.49 ± 3.52 ^cde^	5.24–19.98	8.91 ± 3.24 ^cde^	4.83–16.39	8.26 ± 3.02 ^abe^	5.25–14.84	7.76 ± 2.64 ^ab^	4.72–12.07	7.67 ± 2.60 ^abc^	4.66–13.19	8.086	<0.001 **
	O-Y *p*	0.002 **		0.013 *		0.020 *		0.007 **		0.040 *			
BB	OG	9.27 ± 5.09 ^bcde^	4.32–5.22	8.62 ± 4.32 ^acde^	4.78–23.94	8.03 ± 4.08 ^ab^	4.54–22.77	8.14 ± 4.48 ^ab^	4.23–23.46	7.83 ± 4.25 ^ab^	4.23–23.22	11.697	<0.001 **
	YG	8.69 ± 3.68	4.54–19.74	8.53 ± 3.55	4.81–18.60	8.30 ± 3.17	5.40–17.33	8.21 ± 3.45	5.37–18.02	8.55 ± 3.89	5.50–21.10	3.005	0.023 *
	O-Y *p*	0.536		0.911		0.731		0.931		0.399			
TB	OG	7.20 ± 2.99	4.77–5.89	7.06 ± 3.05	4.80–6.63	7.10 ± 2.99	4.83–6.81	7.63 ± 4.30	4.86–5.82	7.12 ± 2.97	4.79–6.87	0.864	0.489
	YG	7.25 ± 3.48	4.88–19.15	7.32 ± 3.21 ^c^	4.85–16.57	8.22 ± 4.78 ^b^	4.77–24.95	8.41 ± 6.51	4.81–31.86	7.31 ± 3.50	4.86–20.65	3.424	0.012 *
	O-Y *p*	0.938		0.698		0.187		0.504		0.789			
BR	OG	11.43 ± 4.87	5.70–22.87	11.52 ± 5.23	5.64–23.29	11.23 ± 5.24	5.40–22.12	11.34 ± 5.76	5.02–24.49	11.64 ± 6.41	4.82–26.42	0.913	0.46
	YG	9.95 ± 4.14	5.42–27.39	9.56 ± 3.36	5.50–16.69	9.49 ± 3.59	5.44–19.05	9.28 ± 3.67	5.08–18.81	9.08 ± 3.79	4.76–19.65	0.976	0.425
	O-Y *p*	0.124		0.038		0.067		0.046 *		0.023 *			
ES	OG	4.87 ± 2.14 ^e^	3.09–11.73	4.30 ± 2.16 ^e^	3.05–11.77	4.92 ± 2.15 ^e^	3.06–11.90	5.09 ± 2.15	3.03–11.91	5.10 ± 2.18 ^abc^	3.07–11.92	3.852	0.007 **
	YG	3.66 ± 0.36 ^e^	3.10–4.41	3.70 ± 3.69 ^e^	3.21–4.51	3.72 ± 0.36 ^e^	3.22–4.56	4.02 ± 1.22	3.29–9.69	3.89 ± 0.49 ^abc^	3.26–5.18	3.802	0.007 **
	O-Y *p*	<0.001 **		<0.001 **		<0.001 **		0.007 **		0.001 **			

Note: ^a^ indicates a significant difference in comparison with H1, ^b^ indicates a significant difference in comparison with H2, ^c^ indicates a significant difference in comparison with H3, ^d^ indicates a significant difference in comparison with H4, and ^e^ indicates a significant difference in comparison with H5. *: *p* < 0.05, **: *p* < 0.01. O-Y *p*: Comparison between the OG and YG *p*-values.

**Table 5:** The Spearman correlation of the joint angles and test heights.

	Neck Flexion	Right Shoulder Abduction	Right Elbow Internal Rotation	Right Wrist Abduction
OG	−1.000 **	1.000 **	−1.000 **	1.000 **
YG	−1.000 **	1.000 **	−1.000 **	1.000 **

Note: ** indicates a significant correlation at the 0.01 level (two-tailed).

**Table 6:** Mean, standard deviation, and data range of joint angles (/degree) at different test heights, with repeated measures ANOVA results.

Joint		H1		H2		H3		H4		H5		F	*p*
Neck Flexion	OG	20.05 ± 1.83 ^e^	17.04–22.78	20.57 ± 1.83 ^e^	17.09–22.94	20.92 ± 1.91 ^e^	17.83–23.50	21.12 ± 1.91	17.55–23.47	21.70 ± 2.00 ^abc^	18.49–24.27	7.741	<0.001 **
	YG	20.65 ± 2.90 ^bcde^	15.63–26.87	21.48 ± 3.10 ^ae^	15.78–28.95	21.96 ± 3.56 ^ae^	14.96–30.94	22.34 ± 3.29 ^a^	16.07–30.32	22.89 ± 3.25 ^abc^	17.44–31.04	13.136	<0.001 **
	O-Y *p*	0.504		0.336		0.325		0.227		0.237			
Right Shoulder Abduction	OG	40.64 ± 10.11 ^e^	21.29–53.97	39.63 ± 7.10 ^e^	28.08–50.13	36.59 ± 6.03 ^ae^	27.15–46.00	35.81 ± 4.50 ^e^	25.64–43.69	31.69 ± 5.21 ^abcd^	23.65–39.50	10.098	<0.001 **
	YG	35.64 ± 7.81 ^bcde^	21.10–52.43	31.67 ± 8.29 ^ae^	17.10–45.42	30.07 ± 7.44 ^ae^	16.96–42.52	27.67 ± 8.06 ^a^	13.49–40.50	25.78 ± 6.48 ^abc^	14.10–36.14	6.689	<0.001 **
	O-Y *p*	0.141		0.009 *		0.013 *		0.002 *		0.010 *			
Right Elbow Internal rotation	OG	86.58 ± 11.32 ^bcde^	69.89–109.27	90.68 ± 12.88 ^acde^	72.36–114.80	95.70 ± 12.78 ^ab^	77.37–122.48	98.51 ± 13.48 ^ab^	76.09–127.61	100.53 ± 13.58 ^ab^	79.07–131.19	9.477	<0.001 **
	YG	82.26 ± 12.98 ^bcde^	57.33–101.45	88.09 ± 10.68 ^ade^	66.23–103.12	91.19 ± 12.81 ^ade^	72.61–108.86	98.19 ± 10.54 ^abce^	79.06–112.95	102.42 ± 9.23 ^abcd^	84.39–115.55	15.357	<0.001 **
	O-Y *p*	0.340		0.553		0.342		0.943		0.660			
Right Wrist Abduction	OG	24.05 ± 10.31	4.10– 45.18	23.63 ± 9.78	3.39–47.17	23.23 ± 9.27	6.83–45.39	21.16 ± 9.93	2.78–47.89	19.73 ± 9.60	2.42–45.63	2.456	0.072
	YG	25.01 ± 13.38	0.88–51.18	23.36 ± 11.41	5.81–46.36	23.89 ± 12.17 ^de^	5.54–43.58	21.68 ± 12.92 ^c^	2.92–39.22	20.23 ± 12.76 ^c^	0.03–39.25	2.930	0.041 *
	O-Y *p*	0.828		0.944		0.868		0.903		0.903			

Note: ^a^ indicates a significant difference in comparison with H1, ^b^ indicates a significant difference in comparison with H2, ^c^ indicates a significant difference in comparison with H3, ^d^ indicates a significant difference in comparison with H4, and ^e^ indicates a significant difference in comparison with H5. *: *p* < 0.05, **: *p* < 0.01. O-Y *p*: Comparison between the OG and YG *p*-values.

**Table 7:** The significant difference in overall and body parts RPD between the OG and YG *p*-value.

	Overall	Neck	Shoulder	Low Back	Upper Arm	Forearm	Wrist
OG	<0.001 **	<0.001 **	<0.001 **	0.026 *	<0.001 **	0.165	0.028 *
YG	0.173	<0.001 **	0.124	0.004 *	0.083	0.594	0.095

Note: ** indicates a significant correlation at the 0.01 level (two-tailed). * indicates a significant correlation at the 0.05 level (two-tailed).

## Data Availability

Data is contained within the article.

## Appendix A

**Table A1:** Illustration of the joint angles.

Head and trunk flexion angle			
	a. Head Flexion	b. Neck Flexion	c. Back Flexion
	Vertical Angle between C1 and Head.	Vertical Angle between T1 and C7.	Vertical Angle between T9 and T8.
		
	d. Low Back Flexion	e. Hip Flexion	
	Vertical Angle between L1 and T12.	Vertical Angle between L5 and S1.	
Right arm joint angle			
	f1. Right Shoulder Flexion	f2. Right Shoulder Abduction	f3. Right Shoulder Internal Rotation
	The Angle of forward rotation of the AP axis of the shoulder joint	The Angle of outward rotation of the ML axis of the shoulder joint	The Angle of forward rotation of the shoulder joint about the vertical axis.
		
	g1. Right Elbow Flexion	g2. Right Elbow Abduction	g3. Right Elbow Internal Rotation
	The Angle of forward rotation of the AP axis of the elbow joint	The Angle of forward rotation of the AP axis of the elbow joint	The Angle of forward rotation of the elbow joint about the vertical axis.
		
	h1. Right Wrist Flexion	h2. Right Wrist Abduction	h3. Right Wrist Internal Rotation
	The Angle of forward rotation of the AP axis of the wrist joint	The Angle of axial rotation of wrist joint ML to ulnar side	The Angle of forward rotation of the wrist joint about the vertical axis.

Note: The solid arrows mean axes, dashed arrows mean angular direction.

## Appendix B

**Table A2:** Results of repeated measures ANOVA for joint angles at different test heights.

Joint		H1	H2	H3	H4	H5	F	*p*
a. Head Flexion	OG	1.19 ± 3.40 ^e^	2.15 ± 3.39 ^e^	2.78 ± 3.53 ^e^	3.13 ± 3.56	4.17 ± 3.73 ^abc^	7.060	<0.001 *
	YG	2.34 ± 5.39 ^bcde^	3.87 ± 5.75 ^ae^	4.75 ± 6.61 ^ae^	5.43 ± 6.08 ^a^	6.51 ± 6.02 ^abc^	13.102	<0.001 *
	O-Y *p*	0.490	0.325	0.317	0.216	0.211		
c. Back Flexion	OG	6.93 ± 0.62 ^e^	6.95 ± 0.59 ^e^	7.07 ± 0.65 ^e^	7.18 ± 0.61	7.36 ± 0.65 ^abc^	2.812	0.047 *
	YG	6.35 ± 0.39 ^e^	6.47 ± 0.63	6.58 ± 0.61	6.62 ± 0.55 ^e^	6.84 ± 0.72 ^ad^	3.532	0.020 *
	O-Y *p*	0.005 *	0.041 *	0.042 *	0.013 *	0.047 *		
d. Low Back Flexion	OG	1.45 ± 0.82 ^e^	1.49 ± 0.79 ^e^	1.64 ± 0.87 ^e^	1.80 ± 0.81	2.03 ± 0.87 ^abc^	2.811	0.047 *
	YG	0.69 ± 0.52 ^e^	0.85 ± 0.84	0.99 ± 0.82	1.04 ± 0.74 ^e^	1.33 ± 0.96 ^ad^	3.535	0.020 *
	O-Y *p*	0.005 *	0.041 *	0.042 *	0.013 *	0.047 *		
e. Hip Flexion	OG	3.28 ± 1.85 ^e^	2.42 ± 1.77 ^e^	3.71 ± 1.95 ^e^	4.05 ± 1.82	4.57 ± 1.97 ^abc^	2.810	0.047 *
	YG	1.56 ± 1.17 ^e^	1.93 ± 1.89	2.23 ± 1.84	2.36 ± 1.67 ^e^	3.01 ± 2.17 ^ad^	3.529	0.020 *
	O-Y *p*	0.005 *	0.041 *	0.042 *	0.013 *	0.048 *		
f1. Right Shoulder Flexion	OG	48.19 ± 9.89 ^bcde^	43.70 ± 9.64 ^ade^	39.01 ± 9.70 ^ae^	34.58 ± 8.76 ^ab^	32.37 ± 8.12 ^abc^	15.010	<0.001 *
	YG	42.76 ± 12.97 ^bcde^	37.44 ± 13.32 ^ade^	32.85 ± 12.34 ^ae^	28.43 ± 12.38 ^ab^	26.09 ± 11.44 ^abc^	16.565	<0.001 *
	O-Y *p*	0.208	0.151	0.140	0.128	0.094		
f3. Right Shoulder Internal Rotation	OG	30.57 ± 14.17 ^bcde^	24.30 ± 15.72 ^ade^	20.33 ± 16.14 ^ade^	13.39 ± 14.67 ^abc^	11.09 ± 15.26 ^abc^	8.618	<0.001 *
	YG	11.72 ± 13.71 ^e^	10.40 ± 13.88 ^e^	6.94 ± 10.95	3.41 ± 12.27	1.10 ± 13.88 ^ab^	2.838	0.045 *
	O-Y *p*	<0.001 *	0.016 *	0.013 *	0.053	0.071		
g1. Right Elbow Flexion	OG	70.89 ± 12.02 ^cde^	67.10 ± 12.89 ^de^	62.85 ± 11.83 ^ade^	57.67 ± 9.72 ^abce^	53.47 ± 9.13 ^abcd^	9.831	<0.001 *
	YG	77.66 ± 16.72 ^bcde^	70.49 ± 14.22 ^ade^	68.50 ± 14.09 ^ade^	62.76 ± 14.49 ^abce^	55.89 ± 12.98 ^abcd^	17.968	<0.001 *
	O-Y *p*	0.213	0.500	0.245	0.269	0.559		
g2. Right Elbow Abduction	OG	31.19 ± 8.93	32.22 ± 9.24	32.46 ± 8.22	32.03 ± 7.30	29.56 ± 7.54	2.095	0.112
	YG	44.38 ± 9.86	43.33 ± 10.17	43.84 ± 11.53 ^e^	42.21 ± 10.88 ^e^	39.24 ± 10.39 ^cd^	3.812	0.015 *
	O-Y *p*	<0.001 *	0.004 *	0.004 *	0.005 *	0.007 *		
h1. Right Wrist Flexion	OG	1.69 ± 4.94 ^cde^	3.54 ± 6.07 ^ce^	5.61 ± 6.48 ^ab^	5.60 ± 7.31 ^a^	7.65 ± 6.33 ^ab^	12.680	<0.001 *
	YG	3.97 ± 12.43 ^bcde^	6.96 ± 12.19 ^acde^	9.10 ± 13.55 ^abde^	12.24 ± 12.61 ^abc^	12.84 ± 11.93 ^abc^	24.449	<0.001 *
	O-Y *p*	0.514	0.338	0.376	0.089	0.148		
h3. Right Wrist Internal Rotation	OG	11.24 ± 11.92	11.84 ± 12.08	11.96 ± 12.76 ^de^	9.30 ± 12.30 ^c^	8.92 ± 13.45 ^c^	4.011	0.012 *
	YG	11.54 ± 11.87	11.13 ± 12.81	11.24 ± 12.42	11.57 ± 10.63	10.05 ± 11.40	0.912	0.472
	O-Y *p*	0.945	0.877	0.876	0.592	0.806		

Note: ^a^ indicates a significant difference in comparison with H1, ^b^ indicates a significant difference in comparison with H2, ^c^ indicates a significant difference in comparison with H3, ^d^ indicates a significant difference in comparison with H4, and ^e^ indicates a significant difference in comparison with H5. *: *p* < 0.05. O-Y *p*: Comparison between the OG and YG *p*-values.

## References

[1] Chun M.Y., Cho B.-J., Yoo S.H., Oh B., Kang J.-S., Yeon C. (2018). Association between sleep duration and musculoskeletal pain The Korea National Health and Nutrition Examination Survey 2010–2015. Medicine.

[2] Kinge J.M., Knudsen A.K., Skirbekk V., Vollset S.E. (2015). Musculoskeletal disorders in Norway: Prevalence of chronicity and use of primary and specialist health care services. Bmc Musculoskelet. Disord..

[3] Kenny G.P., Yardley J.E., Martineau L., Jay O. (2008). Physical work capacity in older adults: Implications for the aging worker. Am. J. Ind. Med..

[4] Kim D., Lee S.-J., Kim S.-K., Giddings V.L., Robinson S.R. (2019). Home environmental barriers for low-income elderly renters. J. Archit. Plan. Res..

[5] Fu Y.Y., Chui E.W.T. (2020). Determinants of Patterns of Need for Home and Community-Based Care Services Among Community-Dwelling Older People in Urban China: The Role of Living Arrangement and Filial Piety. J. Appl. Gerontol..

[6] Maguire M., Peace S., Nicolle C., Marshall R., Sims R., Percival J., Lawton C. (2014). Kitchen Living in Later Life: Exploring Ergonomic Problems, Coping Strategies and Design Solutions. Int. J. Des..

[7] Varnai A., Nienhaus A., Groneberg D.A., Ohlendorf D. (2019). Posture of employees exemplified by a hospital canteen kitchen. An objective task analysis. Man. Med..

[8] Carrasquillo V., Armstrong T.J., Hu S.J. (2022). Field Observation of Hospital Food Service Workers and the Relationship between Customer Demand and Biomechanical Stress: A Case Study. Iise Trans. Occup. Ergon. Hum. Factors.

[9] Subramaniam S., Murugesan S. (2015). Investigation of work-related musculoskeletal disorders among male kitchen workers in South India. Int. J. Occup. Saf. Ergon..

[10] Marsot J., Claudon L., Jacqmin M. (2007). Assessment of knife sharpness by means of a cutting force measuring system. Appl. Ergon..

[11] Tirloni A.S., dos Reis D.C., Tirloni S.F., Moro A.R.P. (2020). Exertion Perception When Performing Cutting Tasks in Poultry Slaughterhouses: Risk Assessment of Developing Musculoskeletal Disorders. Int. J. Environ. Res. Public Health.

[12] McGorry R.W., Dempsey P.G., O’Brien N.V. (2004). The effect of workstation and task variables on forces applied during simulated meat cutting. Ergonomics.

[13] Maithani H., Corrales Ramon J.A., Lequievre L., Mezouar Y., Alric M. (2021). Exoscarne: Assistive Strategies for an Industrial Meat Cutting System Based on Physical Human-Robot Interaction. Appl. Sci..

[14] Kishtwaria J., Mathur P., Rana A. (2007). Ergonomic evaluation of kitchen work with reference to space designing. J. Hum. Ecol..

[15] Kirvesoja H., Vayrynen S., Haikio A. (2000). Three evaluations of task-surface heights in elderly people’s homes. Appl. Ergon..

[16] Ward J.S. (1971). Ergonomic techniques in the determination of optimum work surface heights. Appl. Ergon..

[17] Panero J., Zelnik M. (1979). Human Dimension and Interior Space: A Source Book of Design Reference Standards.

[18] Ayoub M.M. (2000). Occupational Biomechanics (3rd ed.) Edited by Don B. Chaffin, Gunnar B. J. Andersson, & Bernard J. Martin 1999, 579 pages, $69.96 New York: John Wiley & Sons, Inc. ISBN: 0–471–24697–2. Ergon. Des..

[19] Jeon W., Ramadan A., Whitall J., Alissa N., Westlake K. (2023). Age-related differences in lower limb muscle activation patterns and balance control strategies while walking over a compliant surface. Sci. Rep..

[20] Smith T.M., Hester G.M., Ha P.L., Olmos A.A., Stratton M.T., VanDusseldorp T.A., Feito Y., Dalton B.E. (2020). Sit-to-stand kinetics and correlates of performance in young and older males. Arch. Gerontol. Geriatr..

[21] Huntley A.H., Zettel J.L., Vallis L.A. (2016). Effect of aging on dynamic postural stability and variability during a multi-directional lean and reach object transportation task. Arch. Gerontol. Geriatr..

[22] Ikezoe T., Mori N., Nakamura M., Ichihashi N. (2011). Age-related muscle atrophy in the lower extremities and daily physical activity in elderly women. Arch. Gerontol. Geriatr..

[23] Shi X., Zhang F. (2023). Analysis of the Hanging Actions and Operating Heights of Storage Furniture Suitable for the Elderly. Sensors.

[24] Valipoor S., Pati D., Stock M.S., Bazuin D. (2018). Safer chairs for elderly patients: Design evaluation using electromyography and force measurement. Ergonomics.

[25] Giorgianni C., Principato F., Spatari G. (2023). Upper Limb Disorders in Catering Workers. Diseases.

[26] Abdelsalam A., Wassif G.O.O., Eldin W.S., Abdel-Hamid M.A.A., Damaty S.I.I. (2023). Frequency and risk factors of musculoskeletal disorders among kitchen workers. J. Egypt. Public Health Assoc..

[27] Tegenu H., Gebrehiwot M., Azanaw J., Akalu T.Y. (2021). Self-Reported Work-Related Musculoskeletal Disorders and Associated Factors among Restaurant Workers in Gondar City, Northwest Ethiopia, 2020. J. Environ. Public Health.

[28] Pontonnier C., de Zee M., Samani A., Dumont G., Madeleine P. (2014). Strengths and limitations of a musculoskeletal model for an analysis of simulated meat cutting tasks. Appl. Ergon..

[29] Samani A., Pontonnier C., Dumont G., Madeleine P. (2015). Shoulder Kinematics and Spatial Pattern of Trapezius Electromyographic Activity in Real and Virtual Environments. PLoS ONE.

[30] Cregg A.C., Foley R.C., Livingston L.A., La Delfa N.J. (2021). A biomechanical evaluation of different footrest heights during standing computer work. Ergonomics.

[31] Merbah J., Jacquier-Bret J., Gorce P. (2020). Effect of the presence or absence of upper limb support on posture when a smartphone user is in a seated position under ambient light conditions. Int. J. Ind. Ergon..

[32] Cardoso M.R., Armstrong D.P., Fischer S.L., Albert W.J. (2024). Differential effects of sex on upper body kinematics and kinetics during fatiguing, Asymmetric lifting. Appl. Ergon..

[33] Laksono P.W., Matsushita K., bin Suhaimi M.S.A., Kitamura T., Njeri W., Muguro J., Sasaki M. (2020). Mapping Three Electromyography Signals Generated by Human Elbow and Shoulder Movements to Two Degree of Freedom Upper-Limb Robot Control. Robotics.

[34] Zhou C., Xu X., Huang T., Kaner J. (2024). Effect of different postures and loads on joint motion and muscle activity in older adults during overhead retrieval. Front. Physiol..

[35] Komisar V., Maki B.E., Novak A.C. (2019). Effect of handrail height and age on the timing and speed of reach-to-grasp balance reactions during slope descent. Appl. Ergon..

[36] Jakob M., Liebers F., Behrendt S. (2012). The effects of working height and manipulated weights on subjective strain, body posture and muscular activity of milking parlor operatives–laboratory study. Appl. Ergon..

[37] Human Dimensions of Chinese Adults.

[38] Hermens H.J., Freriks B., Disselhorst-Klug C., Rau G. (2000). Development of recommendations for SEMG sensors and sensor placement procedures. J. Electromyogr. Kinesiol..

[39] Criswell E. (2011). Cram’s Introduction to Surface Electromyography.

[40] Xie Y., Szeto G.P.Y., Dai J., Madeleine P. (2015). A comparison of muscle activity in using touchscreen smartphone among young people with and without chronic neck–shoulder pain. Ergonomics.

[41] Soderberg G.L. (1992). Selected topics in Surface Electromyography for Use in the Occupational Setting: Expert Perspectives.

[42] Wang W. (2016). Research on the Relationship between Body Parameters and sEMG Indicators of STS. Ph.D. Thesis.

[43] Falou W.E., Duchene J., Grabisch M., Hewson D., Langeron Y., Lino F. (2003). Evaluation of driver discomfort during long-duration car driving. Appl. Ergon..

[44] Potvin J.R. (1997). Effects of muscle kinematics on surface EMG amplitude and frequency during fatiguing dynamic contractions. J. Appl. Physiol..

[45] (2000). Ergonomics—Evaluation of Static Working Postures.

[46] McAtamney L., Corlett E.N. (1993). RULA: A survey method for the investigation of work-related upper limb disorders. Appl. Ergon..

[47] Borg G. (1982). Psychophysical based of perceived exertion. Med. Sci. Sport Exerc..

[48] Pheasant S. (2018). Bodyspace: Anthropometry, Ergonomics and the Design of Work.

[49] Mccormick E.J., Sanders M.S. (1992). Human Factors in Engineering and Design.

[50] Balendra N., Langenderfer J.E. (2017). Effect of hammer mass on upper extremity joint moments. Appl. Ergon..

[51] Norheim K.L., Samani A., Madeleine P. (2021). The effects of age on response time, accuracy, and shoulder/arm kinematics during hammering. Appl. Ergon..

[52] Jonsson B. (1978). Kinesiology: With special reference to electromyographic kinesiology. Electroencephalogr. Clin. Neurophysiol. Suppl..

[53] Arjunan S.P., Kumar D.K. (2013). Age-associated changes in muscle activity during isometric contraction. Muscle Nerve.

[54] Hortobágyi T., Mizelle C., Beam S., DeVita P. (2003). Old adults perform activities of daily living near their maximal capabilities. J. Gerontol. Ser. A-Biol. Sci. Med. Sci..

[55] Tikkanen O., Sipila S., Kuula A.-S., Pesola A., Haakana P., Finni T. (2016). Muscle activity during daily life in the older people. Aging Clin. Exp. Res..

[56] Veiersted K.B., Westgaard R.H. (1993). Development of trapezius myalgia among female workers performing light manual work. Scand. J. Work Environ. Health.

[57] Qin J., Lin J.-H., Buchholz B., Xu X. (2014). Shoulder muscle fatigue development in young and older female adults during a repetitive manual task. Ergonomics.

[58] Gheno R., Cepparo J.M., Rosca C.E., Cotten A. (2012). Musculoskeletal Disorders in the Elderly. J. Clin. Imaging.

[59] Kawanishi M., Yahagi S., Shimura K., Kasai T. (1999). Dependence of deltoid muscle activity upon initial angles of shoulder abduction prior to flexion. Percept. Mot. Ski..

[60] Saito H., Yokoyama H., Sasaki A., Kato T., Nakazawa K. (2022). Evidence for basic units of upper limb muscle synergies underlying a variety of complex human manipulations. J. Neurophysiol..

[61] Umehara J., Yagi M., Hirono T., Ueda Y., Ichihashi N. (2021). Quantification of muscle coordination underlying basic shoulder movements using muscle synergy extraction. J. Biomech..

[62] Maeda K., Suenaga T., Churei M., Miyao M. (1986). A factor-control study on disorders of the back, shoulders, neck and upper limbs of cooks. J. Sci. Labour.

[63] Subramaniam S., Murugesan S., Jayaraman S. (2017). Assessment of shoulder and low back muscle activity of male kitchen workers using surface electromyography. Int. J. Occup. Med. Environ. Health.

[64] Pekkarinen A., Anttonen H. (1988). The effect of working height on the loading of the muscular and skeletal systems in the kitchens of workplace canteens. Appl. Ergon..

[65] Sulaiman R., Taha Z., Dawal S.Z.M. (2013). Application of Anthropometric Dimensions for Estimating Stove Height, Stove Depth and Cooking Task Envelope for Malaysian Elderly Population. Pertanika J. Sci. Technol..

[66] Iwakiri K., Kunisue R., Sotoyama M., Udo H. (2008). Postural Support by a Standing Aid Alleviating Subjective Discomfort among Cooks in a Forward-bent Posture during Food Preparation. J. Occup. Health.

